# Supplementary material for: WordCommentsAnalyzer: A windows software tool for qualitative research
Source: F1000Res. 2018 Sep 4;7:536. Originally published 2018 May 3. [Version 2] doi: 10.12688/f1000research.14819.2 (PMC6137411; doi:10.12688/f1000research.14819.2)
Supplement: Supplementary file 3 [file f1000research-7-17614-s0002.tgz › b2a79496-4ada-47c4-9682-a99ce867836d.zip!/Eight Iranian Health Journals/The Journal of Toloo-e-Behdasht_7.docx]

© The Journal of Toloo-e-Behdasht. This work is licensed under CC BY 4.0 (https://creativecommons.org/licenses/by/4.0/).

Volume & Issue: دوره 16 - شماره 1

Title: مقایسه کارایی مدلهای رگرسیون پواسن تعمیم یافته با رگرسیون پواسن استاندارد در تحلیل رفتار باروری زنان شهر کاشان درسال 1391

http://tbj.ssu.ac.ir/article-1-662-fa.html

مقدمه: درتحلیل داده­های باروری از روشهای مختلف آماری می­توان استفاده نمود. زمانی که متغیر پاسخ گسسته باشد، مدل پواسن استفاده می­شود. درصورتی که شرایط مدل پواسن برقرار نباشد،­ ازتعمیم ­یافته ­ی آن استفاده می ­شود.هدف از این مطالعه، مقایسه­ی برازندگی مدل تعمیم یافته نسبت به مدل پواسن معمولی در برآورد ضرائب عوامل موثر برتعداد فرزندان موجود می­باشد. روش بررسی:این مطالعه مقطعی بوده و جامعه­ی موردمطالعه، زنان ازدواج کرده­ی 49-15 شهر کاشان می­باشند. روش انتخاب نمونه به صورت خوشه­ای و خوشه­های مورد نظربلوک­ های شهری بودند که توسط شهرداری تعیین شده بود. تعداد 10خوشه، هر خوشه30 خانوار براساس چارچوب مرکزبهداشت جمع­ آوری گردید واطلاعات لازم ازطریق پرسشنامه­ی خودساخته و یا مصاحبه­ی مستقیم با زنان توسط مراجعه به درب منازل آنها تکمیل گردید. سپس تحلیل داده­ها با  استفاده ازرگرسیون پواسن معمولی و تعمیم یافته توسط نرم افزار R انجام شد. یافته ­ها:میانگین فرزندان هرزن 45/1 و واریانس آن 073/1 است. سن همسر، تعداد حاملگی ناخواسته و متوسط طول مدت شیردهی در دو مدل پواسن معمولی و پواسن استاندارد با تعداد فرزندان موجود رابطه معنی­ دار داشتند (05/.< p). میانگین سنی زنان شرکت کننده دراین مطالعه 57/7 1/33 سال (53/25تا67/40 سال) و میانگین سنی ازدواج زنان 82/3 09/20 سال (27/16 تا91/23 C.I,5%:) و میانگین سنی همسران 4/8 9/37 سال (5/29 تا3/46 C.I,5%:) بود. همانطورکه ملاحظه  می­شود بیشترزنان درگروه سنی 35-30 سال و میانه­ ی سنی آنها 32 سال و بیشتر مردان درگروه سنی 40-35 سال و میانه ­ی سنی آنها 37 سال می باشند. در این مطالعه بیشترزنان دارای 1حاملگی ناخواسته هستند و236 نفر ازآنها حاملگی ناخواسته ندارند. نتیجه­ گیری:نتایج مطالعه نشان دادکه مدل تعمیم ­یافته نسبت به مدل پواسن معمولی جهت تحلیل داده­ ها مناسب­تر می­ باشد ومی تواند ضرائب عوامل موثر بر تعداد فرزندرا دقیق­ تر برآورد نماید.

Title: عنوان: بررسی اثر مکمل یاری بره موم زنبور عسل ( پروپولیس) بر مقاومت انسولینی در بیماران مبتلا به دیابت نوع 2

http://tbj.ssu.ac.ir/article-1-1664-fa.html

مقدمه: دیابت ملیتوس یکی از بیماری های شایع غدد درون ریز است. تعداد مبتلایان به دیابت در طی بیست سال اخیر، دو برابر شده است. آسیا در نتیجه رشد اقتصادی سریع، به عنوان مرکز اپیدمی در جهان مطرح است. ایران نیز جزو کشورهایی است که شیوع دیابت ملیتوس بالایی دارد. استفاده از گیاهان دارویی همیشه به عنوان درمان کمکی در کنار دارودرمانی اصلی مطرح بوده است. در سال های اخیر تمایل بیماران به درمان های جاگزین و طب سنتی بیشتر شده است. روش بررسی: از میان بیماران مراجعه کننده به کلینیک های وابسته به دانشگاه علوم پزشکی شهید صدوقی یزد، 67 نفر انتخاب شده و به صورت تصادفی در دو گروه مداخله یا دارونما قرار گرفتند. بیماران در گروه مداخله روزانه 3 عدد قرص 300 میلی گرمی بره موم زنبور عسل و در گروه کنترل دارونما دریافت کردند. مطالعه 12 هفته به طول انجامید. انسولین سرم و شاخص های مقاومت انسولین در ابتدا و انتهای مطالعه مورد ارزیابی قرار گرفت. یافته ها: 57 نفر مطالعه را به پایان رساندند. میانگین متغیرهای دموگرافیک، شاخص های تن سنجی،انسولین سرم و شاخص های مقاومت به انسولین در آغاز و پایان مطالعه بین دو گروه تفاومت معنی داری را نشان نداد. نتیجه گیری: در مطالعه حاضر، مکمل یاری با بره موم زنبور عسل به مدت 12 هفته، بر شاخص های مقاومت انسولینی بیماران مبتلا به دیابت نوع دو موثر نمی باشد. برای حصول نتیجه قطعی مطالعات بیشتری مورد نیاز است.

Title: بررسی تاثیر عوامل مختلف بر روی کیفیت زندگی زنان نخست زا شهرستان نوشهر1394

http://tbj.ssu.ac.ir/article-1-1650-fa.html

ممقدمه:دوره بعد از زایمان با تغییرات مهمی در کیفیت زندگی زنان همراه می باشد که  می تواند بر سلامت مادر و کودک موثر باشد. این مطالعه با هدف بررسی کیفیت زندگی زنان نخست زا  شهرستان نوشهر انجام شد. روش بررسی: این مطالعه توصیفی- تحلیلی  روی 120 نفر اززنان نخست زا مراجعه کننده به مراکز بهداشتی ودرمانگاههای شهرستان نوشهرکه به طور تصادفی انتخاب شده بودند انجام شدو از ابزار کیفیت زندگی              خلا صه شده سازمان بهداشت جهانی در حیطه های جسمانی، روانی، اجتماعی، محیطی و سلامت عمومی  و نرم افزارSPSS  و  آمار توصیفی و آزمون هایAnova ، t-testو Mann-whithnyبرای تجزیه و تحلیل  استفاده شد. یافته ها:یافته ها  نشان داد  از   حیطه های  مورد بررسی حیطه  اجتماعی با میانگین38/13±56/72 بیشترین نمره و حیطه محیطی با میانگین 29/8±03/64 کمترین نمره را داشتند.مقایسه کیفیت زندگی بر اساس تحصیلات،نوع زایمان،جنسیت نوزاد،شغل  و سن انجام گرفت.از نظر تحصیلات اختلاف معنی داری در حیطه اجتماعی  وجود دارد (05/0P<  )ولی در سایر حیطه ها اختلاف معنی دار نبود.از نظر نوع زایمان اختلاف معنی داری در حیطه اجتماعی و محیطی مشاهده گردید (05/0P<)از نظر جنسیت نوزاد اختلاف معنی داری در حیطه اجتماعی وجود دارد(05/0P< )از نظر نوع شغل اختلاف معنی داری در حیطه اجتماعی مشاهده شد(05/0P<  )از نظر سن اختلاف معنی داری (05/0P<)در حیطه های اجتماعی و محیطی مشاهده شد. نتیجه گیری:بر اساس نتایج حاصل از این پژوهش ملاحظه می گردد که عوامل مختلفی از جمله تحصیلات،نوع زایمان،جنسیت نوزاد،نوع شغل و سن بر کیفیت زندگی زنان نخست زا پس از زایمان  تاثیر دارند و با دانستن این مطلب مراقبین بهداشتی و مسؤلین می توانند گامهای مثبتی در راستای بهبود کیفیت زندگی زنان نخست زا بردارند.

Title: بکارگیری مدل مخاطرات متناسب کاکس در شناسایی عوامل موثر بر بقای بیماران مبتلا به متاستاز مغزی

http://tbj.ssu.ac.ir/article-1-1232-fa.html

چکیده مقدمه : هدف از این مطالعه شناسایی عوامل خطر در بیماران مبتلا به متاستاز مغزی و تعیین عوامل پیش آگهی موثر بر بقای بیماران با استفاده از مدل مخاطرات متناسب کاکس می‌باشد. روش بررسی : این مطالعه توصیفی - تحلیلی گذشته نگر بر روی 197 بیمار مبتلابه متاستاز مغزی مراجعه کننده به مرکز پرتو درمانی شهید رمضانزاده یزد انجام شد. عوامل خطر در این بیماران با استفاده از مدل کاکس مورد تجزیه قرار گرفت. زمان‌های بقاء با استفاده از روش کاپلان - مایر برآورد شد و تفاوت معنی‌دار بین توزیع‌های بقاء در زیر گروه‌های مختلف یک متغیر، توسط آزمون لگ - رنگ مقایسه شدند. آنالیز داده‌ها توسط نرم افزارهای R و SPSS انجام گرفت. یافته‌ها : برآورد کاپلان – مایر بقای کلی بیماران در 12 ماه 27 % و در 24 ماه 12 % بود. با استفاده از آزمون لگ - رنک معلوم شد که تفاوت‌ها بین بقاء بیماران در زیر گروه‌های متغیر سن هنگام تشخیص، بیماری خارج مغزی ( تومور اولیه ) ، نوع درمان، پاسخ به رادیوتراپی و رادیوتراپی مجدد معنی دار بودند (05/0>p). تجزیه و تحلیل با مدل کاکس نشان داد که تنها متغیرهای بیماری خارج مغزی و پاسخ به رادیوتراپی با بقاء بیماران مبتلا به متاستاز مغزی در ارتباط هستند. نتیجه‌گیری : با توجه به نتایج این بررسی، کنترل تومور اولیه و درمان مناسب به وسیله رادیوتراپی جهت افزایش طول عمر بیماران موثر به نظر می‌رسد. همچنین استفاده از مدل کاکس به دلیل تفسیر راحت تر و محدود نبودن استفاده از انواع متغیرهای کمکی در مطالعات پزشکی مناسب است.

Title: بررسی کارایی پرتوهای پرانرژی الکترونی در حذف اسید هیومیک از محلول های آبی

http://tbj.ssu.ac.ir/article-1-280-fa.html

مقدمه: اسید­های هیومیک به­علت اثرات­مضر ناشی از حضور در آب حائز­اهمیت بوده و تلاش­های زیادی جهت حذف این مواد قبل از مرحله گندزدایی انجام شده است. هدف این مطالعه بررسی کارایی پرتوهای پرانرژی الکترونی در حذف اسیدهیومیک از محلول­های آبی است. روش­ بررسی: در این مطالعه تجربی که در مقیاس آزمایشگاهی انجام شد از اسیدهیومیک محصول شرکت سیگما آلدریچ استفاده و بعد از تهیه محلول استوک در شرایط قلیایی، غلظت­های mg/L 10، 25 و50 اسیدهیومیک تهیه شد. در این مطالعه، اثر میزان دز­جذبی 1، 3، 6، 9 و 15 کیلوگری بر حذف اسیدهیومیک در pH 8 بررسی گردید. سپس جذب آن­ها قبل و بعد ازپرتودهی در طول­موج 254 نانومتر با استفاده از دستگاه اسپکتروفتومتر UV-Visible سنجش شد. پس از انجام آزمایش­ها، از نرم­افزارهای Excel و SPSS برای تجزیه وتحلیل داده­ها و رسم نمودارها استفاده گردید. یافته­ها: بررسی اثر pH(10-4)بر کارایی پرتوهای پرانرژی الکترونی در حذف اسیدهیومیک نشان داد که راندمان حذف در pH های مختلف دارای تغییرات جزئی است و بیشترین راندمان حذف در 8=pH مشاهده شده و اختلاف معنادار­آماری­(05/0 ≤P) بین مقادیر راندمان در pHهای مختلف مشاهده نگردید. نتایج مطالعه حاضر نشان داد که با افزایش دز­جذبی پرتو از 1 به 15 کیلوگری، راندمان حذف اسیدهیومیک افزایش و با افزایش غلظت اسیدهیومیک از mg/L 10 به 50، راندمان­حذف کاهش می­یابد. سینتیک واکنش تجزیه اسیدهیومیک در حضور پرتوهای پرانرژی الکترونی با سینتیک شبه­درجه­دوم مطابقت دارد. نتیجه گیری: از این مطالعه می­توان نتیجه گرفت که پرتودهی با پرتوهای پرانرژی الکترونی، تکنولوژی مناسبی برای تصفیه نمونه­های محیطی آلوده­شده توسط اسیدهیومیک می­باشد.

Title: بررسی فعالیت ضد باکتریایی نانو ذرات نقره علیه باکتری‌های گرم مثبت و گرم منفی در محیط آزمایشگاهی

http://tbj.ssu.ac.ir/article-1-1636-fa.html

مقدمه: افزایش مقاومت به عوامل ضد میکروبی معمول یکی از مشکلات عمده بخش مراقبت‌های بهداشتی است. کنترل انتشار باکتری‌ها در محیط‌های مختلف یک چالش موجود در این بخش‌ها است که غلبه بر آن‌ها ازنظر بهداشتی و اقتصادی اهمیت زیادی دارد. در این مطالعه فعالیت ضد باکتریایی نانو ذرات نقره علیه دو باکتری گرم مثبت استافیلوکوکوس اورئوس و سودوموناس آئروژینوزا و دو باکتری گرم منفی اشرشیاکلی و باسیلوس سرئوس بررسی شد. روش بررسی: خاصیت ضد باکتریایی نانو ذرات نقره علیه باکتری‌ها با استفاده از روش‌ رقیق‌سازی مطالعه شد. آزمایش‌های باکتریولوژی با استفاده از غلظت اولیه CFU/ml (108×5/1-1) از هر نوع باکتری‌ انجام شد. مقدار حداقل غلظت ممانعت کننده رشد (MIC) و حداقل غلظت باکتری ‌کشی (MBC) برای هر باکتری در محیط کشت آگار تعیین گردید. زمان مرگ باکتری با استفاده از غلظت­های یک و دو برابر MIC در زمان اثر صفر تا 540 دقیقه بررسی و زمان مرگ هر باکتری تعیین شد. یافته­ها: نتایج نشان داد که حداقل غلظت مهارکنندگی برای باکتری‌های اشرشیاکلای، باسیلوس سرئوس، استافیلوکوکوس اورئوس و سودوموناس آئروژینوزا به ترتیب در غلظت 1700، 1600، 1500 و 1600 میکرو‌گرم در لیتر نانوذره نقره حاصل شد. همچنین، کمترین زمان مرگ برای باکتری‌های باسیلوس سرئوس به مدت 4 ساعت به دست آمد. نتیجه­گیری: بر اساس نتایج حاصل از این مطالعه باکتری‌های اشرشیاکلای کمترین حساسیت و باکتری‌های استافیلوکوکوس اورئوس بیشترین حساسیت را نسبت به نانو ذرات نقره نشان دادند.

Title: مقایسه پروفایل لیپیدی و شاخص های آنتروپومتریک و فشار خون زنان با و بدون استفاده از قرص های با دوز کم پیشگیری از بارداری

http://tbj.ssu.ac.ir/article-1-2429-fa.html

مقدمه: قرص پیشگیری از بارداری یک روش پیشگیری پذیرفته شده در سراسر جهان است. از زمان معرفی این قرص ها استفاده از آنها باعث افزایش خطر عوارض وریدی و شریانی می شود اما در این که آیا قرص های ضد بارداری با دوز کم استروژن (Low Dose) می توانند عامل خطر باشد تردیدهایی وجود دارد. مطالعه حاضر با هدف بررسی مقایسه پروفایل لیپیدی و شاخص های آنتروپومتریک و فشار خون زنان با و بدون استفاده از قرص های با دوز کم پیشگیری از بارداری انجام پذیرفت. روش بررسی: در این مطالعه کوهورت مرکب که با مشارکت 100 خانم سنین 35-20 سال مراجعه کننده به مراکز بهداشتی یزد انجام شد، خصوصیات دموگرافیک به صورت رودررو از طریق مصاحبه توسط محقق پرسیده شده و خصوصیات آنتروپومتریک اندازه گیری و سطح پروفایل لیپیدی با آزمایش بر نمونه های خونی تعیین شده است. سپس داده‌ها با استفاده از نرم افزار  SPSS نسخه 21 و آزمون آماری t-test  و کای اسکوئر تجزیه و تحلیل شده است. یافته ها: در مصرف کنندگان قرص انحراف معیار  ±میانگین مقادیر سرمی کلسترول( mg/dl28/38±7/180)،  تری گلیسرید (mg/dl 92/47±82/129) و  mg/dl)66/30±42/101LDL ( وmg/dl) 42/8±46/56  HDL (و در گروه غیرمصرف کننده کلسترول(mg/dl 26/30±74/)159، تری گلیسرید(mg/dl 01/44±60/93)،  mg/dl) 70/24±84/84 ) LDL و  (mg/dl 91/8 ± 18/56)HDL  بدست آمد. در گروه مصرف کننده در مقایسه با گروه غیرمصرف کننده کلسترول)003/0p=)، تری گلیسرید  ) 000/0p=) و LDL ( 004/0p=) افزایش معناداری نشان داد. بحث و نتیجه گیری: بر اساس نتایج این مطالعه مصرف قرص LD می تواند باعث افزایش سطح کلسترول، تری گلیسرید و LDL شود بنابراین مصرف این قرص می تواند موجب دیس لیپیدمی گردد

Title: بررسی سبک زندگی مربیان بسیج و عوامل مرتبط با آن در دانشگاه های علوم پزشکی استان تهران درسال 93-92

http://tbj.ssu.ac.ir/article-1-1415-fa.html

مقدمه: برنامه بسیجی الگوی زندگی سالم جهت توسعه همکاری های بین بخشی در دفتر آموزش و ارتقای سلامت وزارت بهداشت تدوین و افرادی به عنوان مربیان بسیج انتخاب شدند. بنابراین مطالعه حاضر با هدف تعیین اثر بخشی این مداخله  انجام شد. روش بررسی: مطالعه حاضر، از نوع توصیفی- تحلیلی می باشد که 200 نفر از مربیان سازمان بسیج تحت پوشش دانشگاه های  علوم پزشکی استان تهران به روش سرشماری انتخاب و در سال 93-92  بررسی شدند.  ابزار گردآوری داده ها، پرسشنامه ای محقق ساخته  بود که  روایی و پایایی آن اندازه گیری گردید. داده ها با استفاده از نرم افزار  SPSS نسخه 15آزمونهای توصیفی و تحلیلی مورد تجزیه و تحلیل قرار گرفتند یافته ها:  در این مطالعه 5/93-5/85 درصد از مربیان بسیج، کتاب های بسیجی الگوی زندگی سالم را خوانده، یا کلاس آموزشی آن را گذرانده و یا خودشان آموزش داده بودند. تفاوت آماری معنی داری بین رفتار تغذیه ای در گروههای مختلف سنی وجود داشت. 004/0 p=  همچنین بین رفتار فعالیت جسمانی و سطوح تحصیلی گروه مخاطب نیز تفاوت آماری معنی داری مشاهده گردید  037/0 p=. نتیجه گیری: با توجه به یافته های پژوهش،برنامه آموزشی منجر به اتخاذ داوطلبانه رفتار های سالم در مربیان شده است یعنی مربیان بسیج با کسب دانش و مهارت های مورد نیاز توانسته اند گام های موثری در ارتقای سبک زندگی خود بردارند. به نظر می رسد در صورت حمایت از طرحهایی مثل این بتوان در انتقال این مهارتها و رفتار های سالم به گروه های هدف موفقیت  بیشتری کسب کرد.

Title: نیازسنجی آموزشی در زنان متاهل شهر اهواز به منظور طراحی برنامه توانمندسازی پیشگیری از خشونت خانگی علیه زنان

http://tbj.ssu.ac.ir/article-1-1819-fa.html

مقدمه: سازمان بهداشت جهانی خشونت خانگی علیه زنان را یک اولویت مهم سلامت اعلام کرده است. این مطالعه با هدف تعیین نیازهای آموزشی زنان متاهل مراجعه کننده به مراکز بهداشتی درمانی شهر اهواز جهت طراحی یک برنامه توانمندسازی برای پیشگیری از بروز خشونت علیه آنها انجام شد.  روش بررسی : این مطالعه مقطعی به صورت دو مطالعه کیفی و کمی طراحی گردید. در مطالعه کیفی، دیدگاه ها و نظرات 30 نفر از زنان متاهل ساکن شهر اهواز در قالب چهار جلسه بحث گروهی متمرکز، جمع آوری و تحلیلشد و در مطالعه کمی، با استفاده از پرسشنامه محقق ساخته ی روا و پایا شده، وضعیت آگاهی، نگرش، خودکارآمدی و عملکرد 320 نفر از زنان متاهل در زمینه خشونت خانگی بررسی گردید.  یافته ها: از بین زنان مورد مطالعه، 79% آگاهی کافی درباره خشونت علیه زنان داشتند. 34% در زمینه خشونت خانگی علیه زنان، دارای نگرش مناسب بودند و بیش تر از 80% آنها اعلام داشتند که وجود خشونت علیه زنان توسط مردان امری بدیهی و طبیعی قلمداد می شود. 89%  افراد مورد مطالعه، عدم خودکارآمدی برای جلوگیری از خشونت خانگی علیه زنان را بیان کردند. همچنین یافته ها رابطه معناداری بین میزان تحصیلات و ازدواج در سنین پایین با رفتار خشونت آمیز را نشان دادند. یافته های کیفی نیز نشان دهنده اشکال مختلفی از اعمال خشونت علیه زنان(جر و بحث، دعوا تا خشونت فیزیکی و کتک زدن) توسط شرکت کنندگان بودند عوامل زمینه ساز خشونت از نظر زنان شرکت کننده در چهار گروه عوامل فرهنگی اجتماعی، عوامل اقتصادی، عوامل قانونی ارائه گردید.   نتیجه گیری: آموزش و اطلاع رسانی مناسب به زنان با بحث گروهی و شفاف سازی در جهت تغییر نگرش و افزایش خودکارآمدی در برابر خشونت خانگی از راهکارهای ضروری نتیجه گیری شد که منجر به تغییر نگرش و افزایش توانمندسازی زنان در مقابل خشونت خانگی می شود.
